# Our Task*Read before Oregon State Dental Association, December 3, 1921. Reprinted from *North Western Journal of Dentistry*.

**Published:** 1922-01

**Authors:** C. V. Littler

**Affiliations:** Albany, Ore.


### OUR TASK*

BY DR. C. V. LITTLER, ALBANY, ORE.

This subject, perhaps, is not one which will appeal to most of us, for it implies work, some of which is not of the most pleasant sort.

The small leaflet recently sent to members of our profession deserved more than a passing glance. It was headed: "Tell the Public What Dentistry Stands For." Just what does dentistry stand for? Had the public made a close study of our literature, during the past five years, the very natural conclusion might have been reached that our profession stands for getting as much money from the public, with as little work and in as short time as possible.

Altogether too much time in dental gatherings and too much space in our dental journals has been taken up with advice and suggestions as to how we might increase our fees.

The man was considered a hero who could display a check or a photographic reproduction of one, representing a sum almost equal to what some of our members received for an entire year's work, but which the "Hero" had received for making just one set of muscle trimmed plates.

Our ideas and ideals concerning the successful conduct of a dental practice would in too many cases suffer from a close inspection by the public.

The burden of the message from many who would be leaders in our profession seems to be: "Work less and charge more!"

The dignity of our calling and the value of the services rendered has been pretty well established in the publicity given through the newspapers and magazines, and

Read before Oregon State Dental Association, December 3, 1921. Reprinted from *North Western Journal of Dentistry.*

few there be who have not heard the gospel of "Better Teeth."

More than ever before, the public has been visiting the dentist and many of us have been meeting the awakened public with a pair of forceps in our hand and blood in our eye.

Devitalized teeth and teeth with the pulp exposed have been extracted by wholesale and we have been rapidly degenerating into just common tinkering mechanics.

We have grown chesty over the muscle trimmed plates, which fit so snugly, and the beautiful bridgework, which is so sanitary and artistic and which substitute so well for the natural teeth, many of which should never have been extracted.

If dentistry stands for anything, it stands for the saving of natural teeth. This, more than the restoration of lost teeth by artificial means, is our big task.

Recently, a dentist made the remark: "I simply have to do a lot of bridge and platework; I can't make enough doing chair work."

Is this just an isolated case or does this attitude, together with the hard work necessary to properly treat and fill root canals, influence us too often, in advising extractions?

Dental services, at present, have the distinction of being both a necessity and a luxury. The public, for reasons stated, realize the necessity of having dental work done, and yet, how many can really afford the luxury?

Dental fees are already too high, instead of too low. This does not mean that we are to lower our fees, but there are ways to decrease the cost to the patient, without lowering our fees. Dr. Howe has perfected a plan by which we may save a certain class of teeth in much less time than it formerly took, and by the adoption of this and the discovery of new methods more teeth can be saved with no greater outlay of time and energy.

We should encourage the idea, already taken up by some states, of training dental nurses, who can perform many of the more simple operations, thus giving the dentist more time for the difficult cases.

The dental colleges are not rushing to the aid and relief of the suffering public by increasing the time necessary for a man to prepare himself for the practice of dentistry. If during the late war, at a time when the shortage of food was a serious menace, the food commission had adopted for their slogan, "Produce less food and prolong the war," they would have been mobbed, and what shall we say at a time like this, when obstacles are put in the way of increasing the number of dental practitioners? The Federal Government should forbid such action at this time and until such time as the supply of dentists more nearly meets the demand.

Shall we have it said of our profession what Dr. Chas. Mayo recently said of the Nurses' Union? Here is what he says: "The Nurses' Union has come to be the most autocratic closed shop in this country."

And in view of the enormous task which we face, and the materialistic spirit which has seemed to dominate our dental literature and our personal attitude, it might not be amiss to quote further from this eminent authority, who, in speaking of the noble calling of the nurse, says: "Nursing is quite as much a matter of the heart as of the head and no amount of scientific training will avail to transform a cold-hearted, materialistic woman into a cheerful and efficient ally of health."

In the beautiful city of Portland there are hundreds—yes, thousands—of boys and girls, with decaying and abscessing teeth, and their little bodies are absorbing poisons, the effect of which can not be even estimated. Think of the handicap and the stunting of body, mind and soul.

And yet, I am informed that the free dental clinic was discontinued because of lack of funds. The city could not give the few thousand dollars which would have prevented suffering and saved the lives of the boys and girls of the city.

On the other hand, Portland votes two million dollars for a big exposition. *One meant* only some suffering and perhaps the death of a few boys and girls, while the other would bring many dollars into the pockets of the tax-payers.*

The price of land will go up and buildings will be erected. All very good and to be desired, but after all, what is the big asset of this city or of this country? It is not houses and land, but the boys and the girls. What wonderful possibilities if we could only properly develop them and place the proper valuation upon them.

Our Uncle Sam has been spending much time and money in organizing "better pig" clubs all over the country, and recently one of our leading magazines had a frontispiece, boosting this important and worthy cause. The picture showed a prize pig and the boy who had raised it.

The pig was a beauty and no doubt deserved the rich prize awarded. He was an illustration of what scientific breeding, feeding and care would do for a one-time razor-back. And the boy was an example of what the lack of care will do or fail to do.

The narrow face and protruding lips and teeth indicated adenoids and probably diseased tonsils. There are millions available to insure better stock and better crops, but how very little is being done to care for the alarming

* Editor's Note.—The essayist was misinformed regarding Portland School Clinics. In justice to the dentists of Portland it is here noted that when support of the clinic was impaired through inaction of city and school authorities in regard to maintenance, the Portland Dental Society in one evening raised over $1,500, this sum being supplemented later by a considerable amount and turned over to the Junior Red Cross, which organization has supervised the school dental clinics for the past year and still maintains them effectively, though compared with the need, the financial support is still inadequate.

conditions found in the mouths of the children of our country.

The most prevalent and probably if the truth were known, the most deadly disease in the world today and one which will call for hard work and hard workers in our profession, if we as dentists are to handle and solve the problem.

Educate the public by all means and prevent, so far as possible, the abscesses and extractions in the days ahead, but what of the cases which need attention right now—TODAY?

The National Health Department of Great Britain is presided over by a member of the cabinet, Sir Christopher Addison, who, writing in the *Dental Record* for February, says:

"In Great Britain there are but five thousand dentists, yet there are 5,750,000 school children, the greater bulk of whom have teeth that need caring for."

The free dental clinic, supported by taxation, seems to be the only way open to handle the matter for the present.

The most apparent benefit in the movement to educate the public is that they will be brought to the proper appreciation of their teeth and will thus be willing to be taxed, that all, the poor and the well-to-do, may be cared for.

Our profession ought to take a strong stand for a square deal for the boys and girls of this country. This will mean a hard fight, for we have already discovered that it is easier to get an appropriation to eradicate disease among the livestock than to raise funds for the physical and moral betterment of our boys and girls. The best we can do is little enough, for in a very few years we will not be saying, "Our Task," but "Their Task," for their shoulders will be under the load.—*Northwestern Journal of Dentistry.*

